# Non-coding RNAs in lung cancer: emerging regulators of angiogenesis

**DOI:** 10.1186/s12967-022-03553-x

**Published:** 2022-08-02

**Authors:** Yajie Liao, Xudong Wu, Mengyu Wu, Yuan Fang, Jie Li, Weiqiang Tang

**Affiliations:** 1 Institute of Pharmacy and Pharmacology, The First People’s Hospital of Chenzhou, Hengyang Medical School, University of South China, Chenzhou, 423000 Hunan People’s Republic of China; 2 Department of Thoracic Surgery, The Third Hospital of Changsha, Changsha, 410035 People’s Republic of China; 3 School of Medicine, Jianghan University, Wuhan, 430056 People’s Republic of China; 4 Organ Transplantation Center, The First Affiliated Hospital, Kunming Medical University, Kunming, 650032 Yunnan People’s Republic of China; 5 Institute of Clinical Medicine, The First Affiliated Hospital, Hengyang Medical School, University of South China, Hengyang, 421001 Hunan People’s Republic of China

**Keywords:** Lung cancer, Angiogenesis, Non-coding RNAs, MicroRNAs, Mechanisms

## Abstract

Lung cancer is the second cancer and the leading cause of tumor-related mortality worldwide. Angiogenesis is a crucial hallmark of cancer development and a promising target in lung cancer. However, the anti-angiogenic drugs currently used in the clinic do not achieve long-term efficacy and are accompanied by severe adverse reactions. Therefore, the development of novel anti-angiogenic therapeutic approaches for lung cancer is urgently needed. Non-coding RNAs (ncRNAs) participate in multiple biological processes in cancers, including tumor angiogenesis. Many studies have demonstrated that ncRNAs play crucial roles in tumor angiogenesis. This review discusses the regulatory functions of different ncRNAs in lung cancer angiogenesis, focusing on the downstream targets and signaling pathways regulated by these ncRNAs. Additionally, given the recent trend towards utilizing ncRNAs as cancer therapeutics, we also discuss the tremendous potential applications of ncRNAs as biomarkers or novel anti-angiogenic tools in lung cancer.

## Background

Lung cancer ranks second in incidence among all tumor types worldwide and causes almost one-fourth of cancer deaths. Non-small cell lung cancer (NSCLC) accounts for more than 80% of lung cancers and is the most common histological type of lung cancer [1, 2]. Owing to the highly aggressive nature of NSCLC and the difficulty in diagnosing it at an early stage, the majority of NSCLC patients already have advanced-stage disease at diagnosis. The standard treatment for advanced NSCLC relies on systemic chemotherapy, targeted therapy, immunotherapy, and radiotherapy [3]. The overall survival of lung cancer patients has improved with advancements in treatment methods and earlier diagnosis in recent decades. However, due to distant metastasis and tumor recurrence after treatment, the outcomes of lung cancer patients are still poor [4].

Angiogenesis refers to the formation of new blood vessels from preexisting capillaries or postcapillary venules and is one of the hallmarks of cancer [5]. Unlike physiological conditions, such as wound healing, tumor angiogenesis is persistently aberrant in growing tumors, as they require oxygen and nutrients delivered through the blood circulation system to survive and proliferate [6]. In addition, cancer cells can enter the blood circulation through angiogenesis, resulting in hematogenous tumor metastasis [7, 8]. Over the past few decades, accumulating evidence has revealed that angiogenesis is a vital cancer hallmark related to poor prognosis in various solid tumors [9, 10]. Anti-angiogenic agents can affect the tumor microenvironment to regress existing tumor vessels while inhibiting tumor angiogenesis [11–13]. However, anti-angiogenic drugs targeting vascular endothelial growth factor (VEGF) or the vascular endothelial growth factor receptor (VEGFR) rarely result in durable responses and have had a limited effect on improving the overall survival of patients with lung cancer [14]. Consequently, to exploit new therapeutics to overcome this poor efficacy, further research is needed to discover the mechanisms of angiogenesis in lung cancer.

Angiogenesis is a complex process regulated by many pro-angiogenic and anti-angiogenic genes, and numerous studies have confirmed that angiogenesis is crucial for lung cancer cell proliferation and metastasis [15]. Accumulating evidence suggests that non-coding RNAs (ncRNAs), especially microRNAs (miRNAs) and long non-coding RNAs (lncRNAs), can be involved in tumor angiogenesis by controlling different genes and pathways, resulting in distant metastasis and tumor recurrence [16]. Vascular homeostasis is managed by many different pro- and anti-angiogenic genes, and VEGF has been identified as a critical gene in inducing lung cancer angiogenesis [17–19]. The binding of VEGF and VEGFR initiates various intracellular signaling pathways and mediates the survival, proliferation, and migration of vascular endothelial cells (ECs), in turn promoting angiogenesis and enhancing vascular permeability in lung cancer [20]. Additionally, VEGF can also be produced by tumor immune cells and regulate the functions of innate and adaptive immune cells towards immunosuppression [21]. Hypoxia is the most crucial inducer of angiogenesis, and hypoxia-inducible factors (HIFs) have a crucial function in physiological adaptation to hypoxic states [22]. HIF proteins, especially HIF-1 alpha (HIF-1α), are closely associated with lung cancer occurrence, metastasis, and angiogenesis [23–25]. Multiple studies have indicated that the activation and expression levels of HIF-1α are closely correlated with the outcome of lung cancer [26, 27]. HIF-1α can target VEGFA and regulate its expression at the transcriptional level. Targeting the HIF-1α/VEGFA axis could be a promising strategy against tumor angiogenesis [28].

**Fig. 1:**
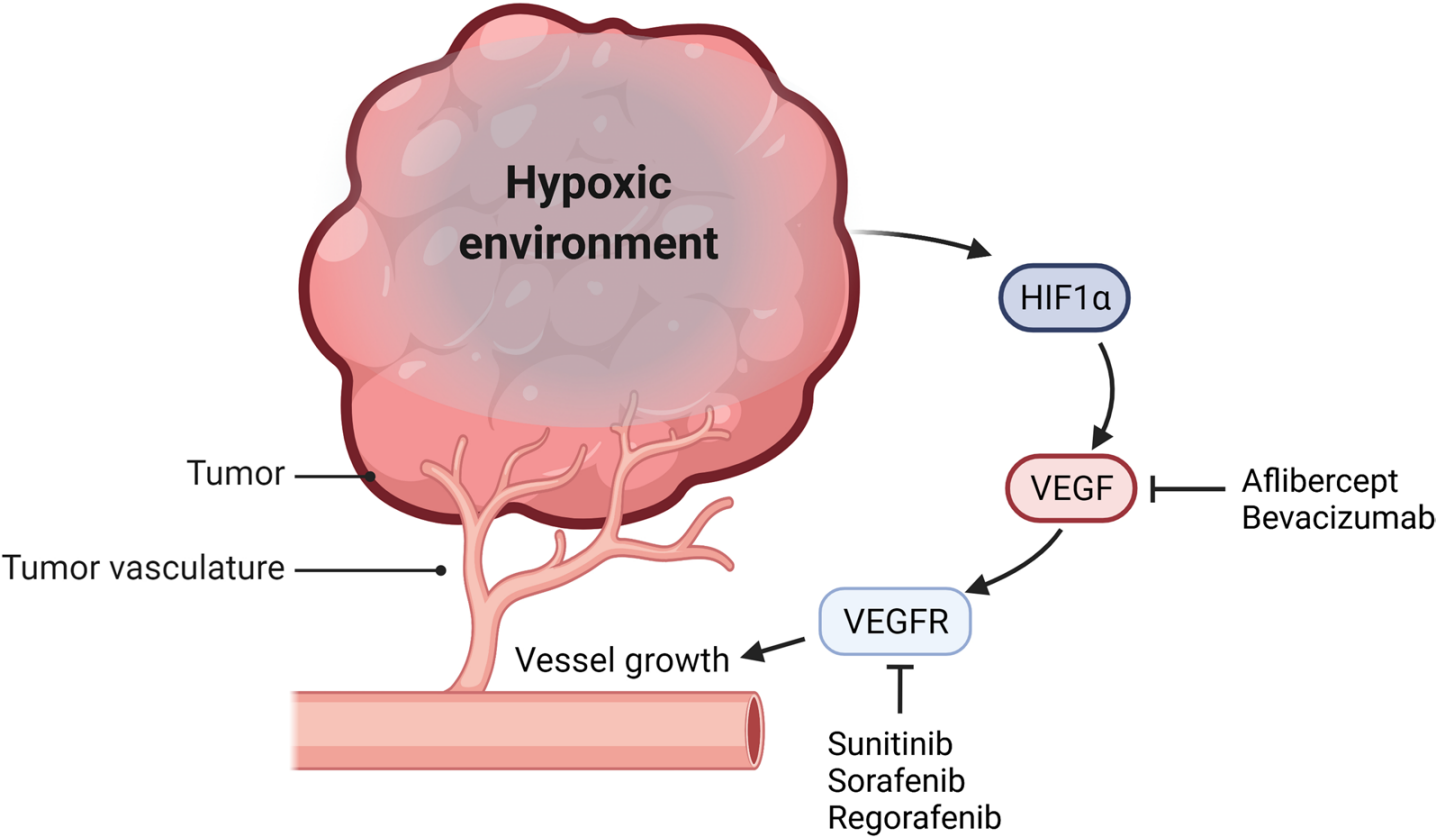
Hypoxia leads to activation of angiogenic signaling in lung cancer. Hypoxia in the tumor microenvironment can regulate angiogenesis in lung cancer by affecting HIF-1 protein expression and regulating the transcription of the downstream target gene VEGF. Created with www.BioRender.com

To date, the US Food and Drug Administration (FDA) has approved various drugs targeting VEGF/VEGFR for tumor anti-vascular therapy (Fig. 1) [29]. Although these drugs have demonstrated efficacy in NSCLC patients, the application of drugs is still affected by clinically significant bleeding events, such as major hemoptysis. On the other hand, long-term VEGF/VEGFR inhibitors probably lead cancer cells to exploit different angiogenic mechanisms and develop drug resistance [30]. Hence, full understanding of the mechanism of tumor angiogenesis will facilitate the discovery of novel therapeutic approaches to improve the prognosis of patients. NcRNAs have become promising biomarkers and potential therapeutic tools in many cancers because of their high stability and good biocompatibility [31–33]. Since ncRNAs act as pro- or anti-angiogenesis factors, their function in angiogenesis in lung cancer can be divided into enhancing or inhibiting, which is recapitulated in Table 1.

**Table 1:** NcRNAs mediating angiogenesis in lung cancer

NcRNAs	Expression	Target gene and pathway	Angiogenic Effect	Type of lung cancer	References
miR-29c	Down-regulation	VEGFA	Inhibits	LUAD	[39]
miR-519c	Down-regulation	HIF-1α/VEGFA axis	Inhibits	NSCLC	[40]
let-7b/miR-126	Down-regulation	VEGFA	Inhibits	NSCLC	[41]
miR-145-5p	Down-regulation	CP	Inhibits	LUAD	[42]
miR-320b	Down-regulation	HNF4G	Inhibits	NSCLC	[43]
miR-497	Down-regulation	HDGF and VEGFA	Inhibits	NSCLC	[51]
miR-141	Up-regulation	KLF12 and GAX	Activates	SCLC, NSCLC	[54, 55]
miR-619-5p	Up-regulation	RCAN1.4	Activates	NSCLC	[56]
LINC00173 V1	Up-regulation	miR-511-5p/VEGFR axis	Activates	LUSC	[60]
lncRNA MCM3AP-AS1	Up-regulation	miR-340-5p/KPNA4 axis	Activates	NSCLC	[61]
linc00665	Up-regulation	YB1/VEGFA axis	Activates	LUAD	[62]
lncRNA TNK2-AS1	Up-regulation	STAT3/VEGFA axis	Activates	NSCLC	[63]
linc00941	Up-regulation	miR-877-3p/VEGFA axis	Activates	NSCLC	[67]
lncRNA F630028O10Rik	Up-regulation	miR-223-3p/VEGFA axis and VEGFR2	Activates	NSCLC	[68]
lncRNA FBXL19-AS1	Up-regulation	miR-431-5p/RAF1 axis	Activates	NSCLC	[69]
lncRNA CCAT1	Up-regulation	N/A	Activates	LUAD	[70]
lincRNA-p21	Up-regulation	N/A	Activates	NSCLC	[72]
circ_0006988	Up-regulation	miR-491-5p/MAP3K3 axis	Activates	NSCLC	[73]
circ_0016760	Up-regulation	miR-29b/HIF1α axis	Activates	NSCLC	[74]
miR-942	Up-regulation	FOXO1	Activates	LUAD	[75]
miR-31	Up-regulation	FOXO3a	Activates	NSCLC	[79]
miR-206	Down-regulation	VEGFA/CCL2	Inhibits	NSCLC	[79]
miR-1	Down-regulation	VEGFA/CCL2 and MPL	Inhibits	NSCLC	[79, 83]
miR-210	Up-regulation	JAK2/STAT3 signaling and TET2	Activates	NSCLC	[79]
miR-224	Up-regulation	SIRT3/AMPK axis	Activates	NSCLC	[80]
miR-103a	Up-regulation	PTEN	Activates	NSCLC	[81]
miR-128	Down-regulation	VEGFC	Inhibits	NSCLC	[82]
miR-200b	Down-regulation	QKI/CCND1 axis	Inhibits	NSCLC	[85]
miR-192	Down-regulation	IL-8, ICAM and CXCL1	Inhibits	LUAD	[86]
miR-494	Up-regulation	PTEN	Activates	NSCLC	[87]
miR-21	Up-regulation	STAT3/VEGF axis	Activates	N/A	[88]
miR-23a	Up-regulation	PHD1 and PHD2	Activates	NSCLC	[89]
lncRNA EPIC1	Up-regulation	Ang2/Tie2 axis	Activates	NSCLC	[91]
lncRNA GAS5	Down-regulation	miRNA-29-3p/PTEN axis	Inhibits	NSCLC	[92]

This table summarizes the dysregulation of miRNAs, lncRNAs, and circRNAs involved in lung cancer angiogenesis and their targets

## Angiogenesis related ncRNAs in lung cancer cells

### Angiogenesis related miRNAs in lung cancer cells

MiRNAs are a class of short single-stranded ncRNA molecules with a length of approximately 22 nucleotides that can regulate gene expression by binding to the 3′ untranslated region (3′ UTR) of mRNAs [34, 35]. Abnormally expressed miRNAs were confirmed to affect various biological processes in cancer, including angiogenesis [36–38].

#### MiRNA directly targets angiogenesis related genes in lung cancer cells

MiRNAs can regulate tumor angiogenesis by directly binding to angiogenesis-related genes. For example, miR-29c has a binding site in the 3' UTR of VEGFA mRNA and can down-regulate the expression level of VEGFA, thereby promoting the capability of tumor cells to induce tube formation of human umbilical vein endothelial cells (HUVECs) in lung adenocarcinoma (LUAD) [39]. MiR-519c can target HIF-1α and reduce the expression of HIF-1α protein, thereby reducing tumor angiogenesis in NSCLC. In addition, hepatocyte growth factor (HGF), an inducer of HIF-1α, suppresses miR-519c maturation through an Akt-dependent pathway, indicating the important role of the HGF/miR-519c/HIF-1α axis in modulating angiogenesis in lung cancer [40]. The let-7 miRNA family is dysregulated in various tumors and participates in various biological processes, such as oncogenesis and development. The expression of let-7b and miR-126 decreases in NSCLC tissues, and their low expression is associated with poor prognosis. Regulation of these two miRNAs reduces tumor angiogenesis and thus inhibits tumor growth in lung cancer patients, which may be a potential treatment for lung cancer [41].

MiR-145-5p can directly target ceruloplasmin (CP), and loss of miR-145-5p in LUAD induces overexpression of CP. Overexpression of CP decreases Fe^2+^ and prolyl hydroxylase domain (PHD) 1/2 levels while inhibiting HIF-2α, leading to the activation of tumor angiogenesis [42]. The expression of miR-320b is down-regulated and related to improved overall survival in patients with lung cancer. The results of gain-of-function experiments suggested that miR-320b can target hepatocyte nuclear factor 4 gamma, thereby inhibiting tumor proliferation, invasion, and angiogenesis in xenografted nude mice [43]. MiR-497 expression has been reported to be significantly decreased in various cancers and acts as a tumor suppressor by targeting different oncogenes [44–47]. MiR-497 was also found to be considerably down-regulated in NSCLC and can suppress angiogenesis, cancer cell proliferation, and invasion by targeting HDGF and VEGFA [48, 49].

#### MiRNAs target angiogenesis related genes in lung cancer cells via exosomes

Microvesicles (MVs) and exosomes can transport ncRNAs and interact with other cells, thus influencing angiogenesis in lung cancer [50]. Jeong et al. demonstrated that exosomes loaded with miR-497 have a synergistic inhibitory effect on targeting the growth and angiogenesis of lung cancer cells and hold promise as a novel approach for tumor-targeted therapy [51]. The miR-200 family inhibits metastasis by regulating tumor angiogenesis in various tumors. Several studies have shown that miR-141, a member of the miR-200 family, exerts pro-angiogenic or anti-angiogenic effects in different cancers [52, 53]. Mao et al. reported that the miR-141 level was significantly increased in serum from patients with small cell lung cancer (SCLC) and that this increase was associated with advanced clinical characteristics. Mechanistically, miR-141 is packaged into exosomes released from SCLC cells and then targets KLF12 and GAX, leading to angiogenesis and malignant progression of lung cancer [54, 55]. A study by Kim et al. reported that NSCLC-derived exosomal miR-619-5p targets RCAN1.4, thereby inducing tumor angiogenesis and metastasis [56]. Fan et al. indicated that miR-210 regulates JAK2/STAT3 signaling and TET2 in recipient fibroblasts, thus initiating the pro-angiogenic switch of cancer-associated fibroblasts (CAFs). Moreover, miR-210 is up-regulated in exosomes released from lung cancer cells, indicating the key role of miR-210 in angiogenesis in lung cancer [57].

### Angiogenesis related lncRNAs in lung cancer cells

LncRNAs are a class of single-stranded RNAs that are longer than 200 nucleotides and have no protein-coding function. LncRNAs can interact with proteins, DNA, and RNA to participate in the regulation of various biological processes. Numerous studies have indicated that dysregulated expression of lncRNAs is associated with the tumorigenesis and progression of multiple cancers, including lung cancer [58, 59].

#### LncRNAs regulate angiogenesis in lung cancer cells through ceRNA networks

LncRNAs can act as ceRNA to regulate gene expression by competitively binding miRNAs. For instance, Chen et al. reported that LINC00173.v1 binds to miR-511-5p to regulate VEGFA expression, thereby promoting angiogenesis and development in lung squamous cell carcinoma (LUSC). Animal experiments demonstrated that LINC00173.v1 increases the therapeutic sensitivity of LUSC cells to cisplatin and that targeting LINC00173.v1 could be a potential treatment for combating LUSC [60]. Li et al. found that the transcription factor YY1 mediates the transcription and expression of lncRNA MCM3AP-AS1 in lung cancer. In addition, MCM3AP-AS1 targets miR-340-5p to induce overexpression of KPNA4, thereby promoting angiogenesis and progression of lung cancer [61]. Linc00941 is a carcinogenic lncRNA and mediates the progression of gastric cancer, head and neck squamous cell carcinoma, and thyroid papillary carcinoma [62–64]. Ren et al. found that linc00941 expression is increased in NSCLC tissues and patient plasma. Additionally, linc00941 interacts with miR-877-3p to regulate VEGFA, accelerating NSCLC angiogenesis and tumor progression [65]. LncRNA F630028O10Rik is a novel lncRNA that can interact with miR‐223‐3p and results in VEGFA and VEGFR2 suppression, thereby regulating tumor angiogenesis and inhibiting tumor growth and progression [66]. Jiang et al. found that lncRNA FBXL19-AS1 expression is increased in lung cancer, and a high level of FBXL19-AS1 expression is related to poor prognosis. Exploration of the molecular mechanism suggested that FBXL19-AS1 participates in the development and angiogenesis in lung cancer by targeting the miR-431-5p/RAF1 axis [67].

#### LncRNAs regulate angiogenesis in lung cancer cells via exosomes

In addition to functioning as miRNA sponges, some lncRNAs can also regulate angiogenesis through exosome transport. LincRNA-p21 is a lncRNA activated by tumor protein p53 and the HIF-1α subunit. Hypoxia can guide lincRNA-p21 expression, which leads to the induction of angiogenesis and correlates with the poor prognosis in NSCLC patients. Meanwhile, NSCLC cell-derived exosomal lincRNA-p21 can promote tube formation of endothelial cells and enhance tumor cell adhesion to endothelial cells [68, 69]. These studies demonstrated the extensive influence of lncRNA-miRNA-mRNA systems in angiogenesis in lung cancer.

#### LncRNAs regulate angiogenesis in lung cancer cells by interacting with proteins

LncRNAs can also interact with proteins to affect the expression of genes. For example, linc00665 can directly interact with the YB-1 protein and accumulate in the nucleaus, thereby increasing ANGPT4, ANGPTL3, and VEGFA expression and promoting tumor-associated angiogenesis in lung cancer [70]. Wang et al. found that lncRNA TNK2-AS1 expression is increased in NSCLC and associated with poor prognosis. Molecular mechanistic studies revealed that the lncRNA TNK2-AS1 interacts with STAT3, increasing its protein stability, and that STAT3 can also bind to the TNK2-AS1 promoter to trigger its transcription. The positive feedback loop promotes the angiogenesis of NSCLC by enforcing STAT3/VEGFA signaling [71]. LncRNA colon cancer-associated transcript 1 (CCAT1) acts as a carcinogenic factor in various tumors and is significantly highly expressed in LUAD. Mechanistic studies revealed that CCAT1 interacts with fatty acid binding protein 5 (FABP5) and mediates the translocation of FABP5 into the nucleus. In addition, CCAT1 can stabilize the PI3K/Akt/mTOR signaling pathway, thus promoting tumor growth and angiogenesis in LUAD [72]. These studies further revealed the effects of lncRNAs on angiogenesis, suggesting that lncRNAs may be an effective target for the treatment of lung cancer.

### Angiogenesis related circRNAs in lung cancer cells

Circular RNA (circRNA) is a novel type of circular ncRNA generated by back-splicing, and their primary function is to act as a sponge for miRNAs to regulate diverse functions of cells [50]. Circ_0006988 can competitively inhibit miR-491-5p, further regulating MAP3K3 and promoting proliferation, metastasis, and angiogenesis in NSCLC cells [73]. Circ_0016760 expression is significantly increased in both NSCLC tissues and cells and can promote the malignant phenotype of NSCLC. Mechanistically, up-regulation of circ_0016760 acts as a sponge of miR-29b and promotes the oncogenic effect of HIF-1α, further inducing malignancy and angiogenesis in NSCLC [74].

There are few studies on the function of circRNAs in lung cancer angiogenesis. However, considering the advantages of circRNAs, such as their low molecular weight and high stability, circRNA-based molecular therapy may be a potential treatment for lung cancer.

## Angiogenesis related ncRNAs in the lung cancer tumor microenvironment

### MiRNAs in the tumor microenvironment affect angiogenesis

Ample evidence indicates that the tumor microenvironment can influence tumor development from multiple aspects, including invasion, metastasis, and angiogenesis [75–77]. Shen et al. compared the miRNA expression profile in CAFs with that in matched NFs from lung cancer patients and found that miR-31 expression was increased in lung CAFs, whereas the expression of miR-1 and miR-206 was increased in the matched NFs. MiR-1, miR-206, and miR-31 can target VEGFA/CCL2 and FOXO3a to regulate their expression. Modulation of the expression of these miRNAs can markedly inhibit tumor angiogenesis, tumor-associated macrophages accumulation, tumor growth and lung metastasis, which may have potential clinical implications for therapy in lung cancer [78]. Zhang et al. indicated that the significant up-regulation of miR-224 in CAFs targeted SIRT3 and thus regulated the SIRT3/AMPK/mTOR/HIF-1 α axis. Furthermore, HIF-1α enhanced the expression of miR-224. This positive feedback loop induced endothelial cell angiogenesis and tumor progression in NSCLC [79].

### Exosomal miRNAs in the tumor microenvironment regulate angiogenesis

Exosomes could perform a communication function between tumor microenvironmental components to regulate angiogenesis in lung cancer. Wei et al. found that M2 macrophage-derived exosomal miR-942 can regulate FOXO1 protein expression by binding to the 3′UTR of FOXO1, thereby promoting LUAD cell invasion and enhancing angiogenesis [80]. Hsu et al. reported that lung cancer-derived EV miR-103a targets phosphatase and tensin homologue deleted on chromosome 10 (PTEN) and enhances M2 polarization to up-regulate the stimulatory effect of macrophages on tumor development and angiogenesis. Mechanistically, miR-103a can directly target PTEN and lead to activation of the PI3K/Akt and STAT3 signaling pathways. Preventing the transfer of EVs from hypoxic cancer cells or suppressing miR-103a expression may be a new therapeutic option to activate the immune response [81].

## Angiogenesis related ncRNAs in the tumor endothelium

### Angiogenesis related miRNAs in the tumor endothelium

#### MiRNAs directly target angiogenesis related genes in tumor endothelial cells

NcRNAs in tumor endothelial cells can participate in lung tumor progression by regulating tumor angiogenesis. Hu et al. found that miR-128 can bind to VEGFC and inhibit tumor growth and invasiveness in vitro. Furthermore, up-regulation of miR-128 in NSCLC cells and HUVECs not only causes down-regulation of VEGFA, VEGFR2, and VEGFR3 but also inhibits angiogenesis and lymphangiogenesis in tumor xenografts [82]. Korde et al. indicated that the expression of miR-1 was decreased in NSCLC, which was related to overall survival. In endothelial cells, miR-1 can regulate the proliferation and angiogenesis of lung cancer cells by binding to the myeloproliferative leukemia virus oncogene [83]. As one of the members of the miR-200 family, miR-200b participates in multiple physiological functions of endothelial cells [84]. MiR-200b inhibits proliferation and sprouting angiogenesis through the QKI/CCND1 axis in endothelial cells and is expected to become a novel target for therapeutic inhibition of NSCLC metastasis [85].

#### miRNAs target angiogenesis related genes in tumor endothelial cells via exosomes

Tumor-derived exosomes can also transport miRNA to endothelial cells to inhibit angiogenesis in lung cancer. NSCLC-derived exosomal miR-192 can target endothelial cells and inhibit tumor angiogenesis and bone metastatic activity by suppressing proangiogenic IL-8, ICAM, and CXCL1 in lung cancer [86]. MiR-494 can be transfected from the lung cancer cell Line A549 into endothelial cells by MVs and promotes angiogenesis mediated by targeting PTEN and subsequently activating the Akt/eNOS pathway [87]. Liu et al. indicated that the miR-21 expression level in the serum of smokers was increased compared with that in non-smokers. In addition, miR-21 can be transferred among human bronchial epithelial (HBE) cells via exosomes, activating STAT3 and consequently up-regulating VEGF levels in recipient cells [88]. Hsu et al. found that miR-23a expression was significantly increased in lung cancer exosomes under hypoxic conditions. Hypoxic lung cancer cell-derived exosomal miR-23a directly binds to PHD1 and PHD2, resulting in HIF-1 accumulation in endothelial cells and enhancing angiogenesis [89].

### Angiogenesis related lncRNAs in tumor endothelium

**Fig. 2:**
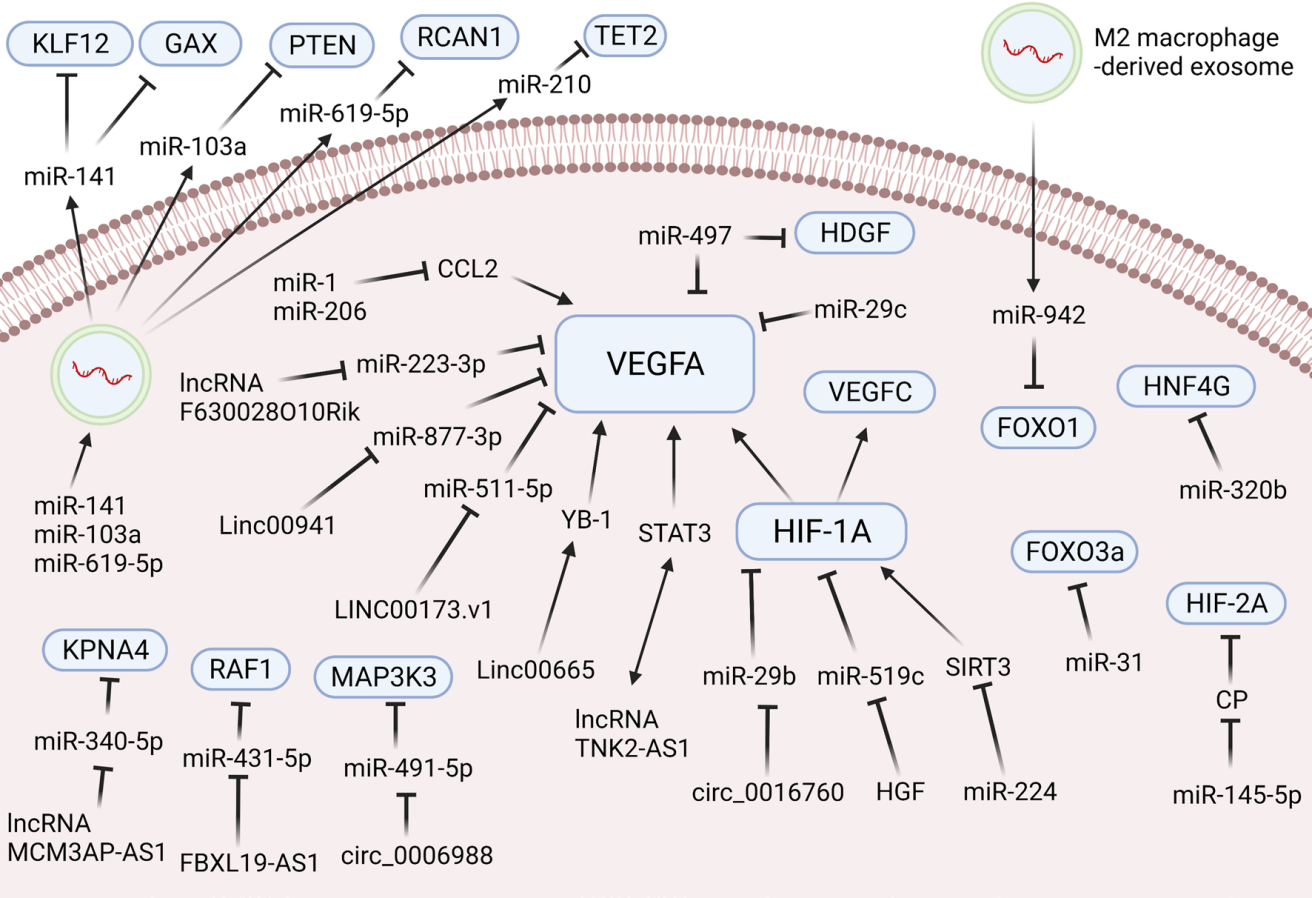
NcRNAs affecting angiogenesis in lung cancer cells and tumor microenvironment cells. NcRNAs can directly or indirectly interact with angiogenesis-related genes in lung cancer cells or the tumor microenvironment, thereby affecting lung cancer angiogenesis. Created with www.BioRender.com

**Fig. 3:**
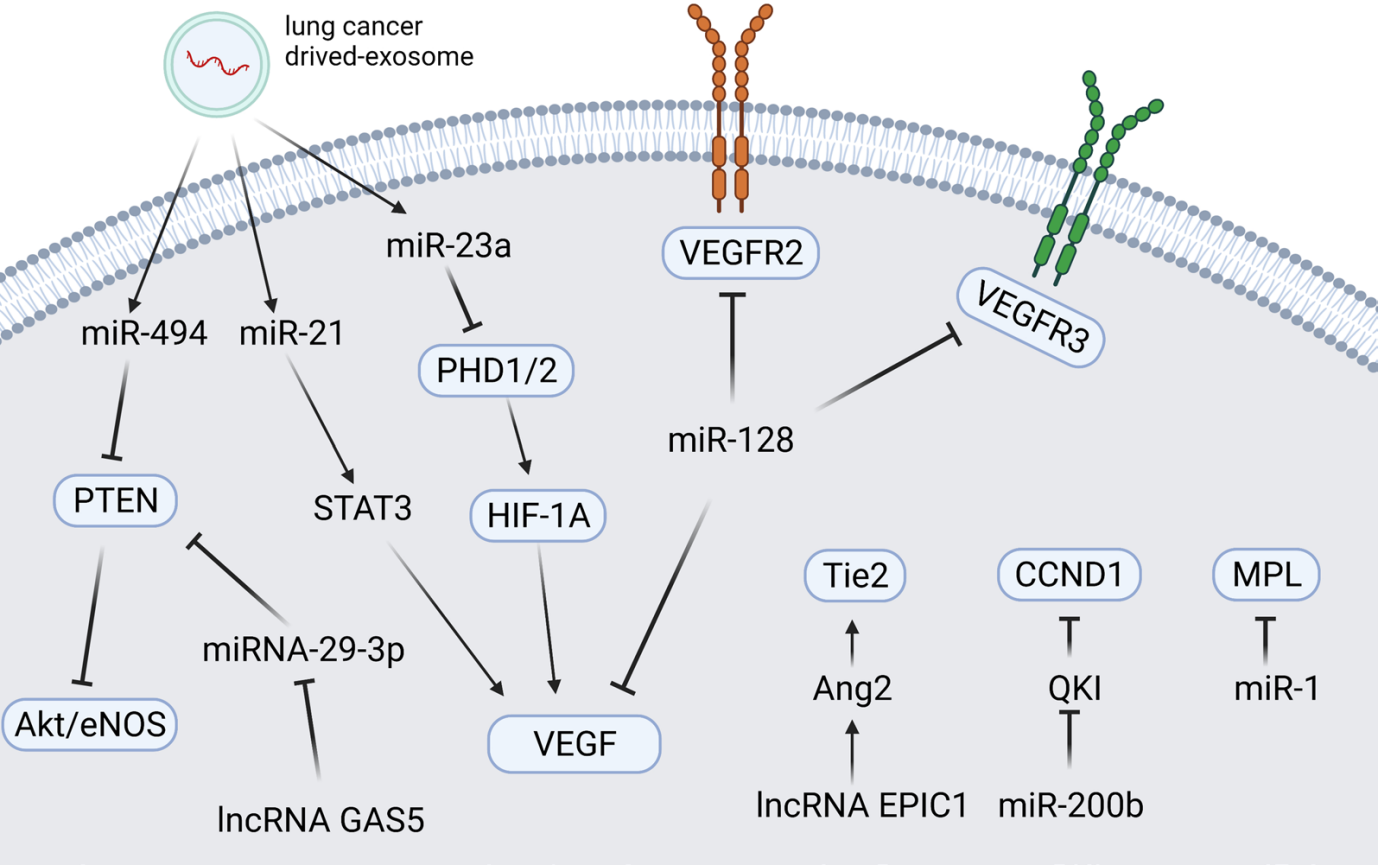
NcRNAs affecting angiogenesis in tumor endothelium cells. In tumor endothelial cells, ncRNAs can act on angiogenic or proliferation-related genes to induce angiogenesis. Created with www.BioRender.com

The lncRNA EPIC1, a MYC-interacting lncRNA, can promote cell-cycle progression in cancer [90]. The lncRNA EPIC1 was significantly up-regulated in NSCLC tissues and cell lines. Animal experiments demonstrated that overexpression of the lncRNA EPIC1 can activate HUVEC channel formation and proliferation by activating the Ang2/Tie2 axis in NSCLC [91]. Cheng et al. pointed out that the lung cancer cell-derived exosomal lncRNA growth arrest-specific transcript 5 (GAS5) competitively targets miRNA-29-3p with PTEN in HUVECs to affect their proliferation, apoptosis, and tube formation [92]. These outcomes demonstrate that regulation of lncRNAs in HUVECs can ameliorate the malignant phenotype of tumors and that lncRNAs could be a potential target in the anti-angiogenic treatment of lung cancer (see Fig. 2, 3).

## Prospects and challenges of ncRNA-based anti-angiogenesis therapy

NcRNAs are crucial regulatory factors in tumorigenesis that activate or inhibit the oncogenic process. Therefore, advancing practical therapeutic approaches to suppress oncogenic ncRNAs or overexpress cancer-associated ncRNAs is becoming a hot research field [93]. Eleven ncRNA-based therapies, all of which are small interfering RNAs (siRNAs) or antisense oligonucleotides (ASOs) targeting specific genes, have been approved for clinical treatment. Moreover, many ncRNA-based therapeutics are in clinical development with potential applications in various tumors, including lung cancer [94]. The ncRNA-based anti-angiogenesis therapies in lung cancer presented in Table 2.

**Table 2:** NcRNA-based anti-angiogenesis therapies in lung cancer

Therapeutic tools	Type	Targets	Functions	References
Nucleolin aptamer-siRNA chimaeras	siRNA	SLUG and NRP1	Suppress lung cancer cell invasion, tumor growth and angiogenesis	[96]
Bivalent cyclic RGD-siRNA conjugate	siRNA	VEGFR2	Inhibiting neovascularization and proliferation on NSCLC xenografts	[97]
siRNA nanoemulsions	siRNA	CXCR4 and STAT3	Inhibit tumor proliferation and neovascularization in lung metastases	[99]
Global miRNA depletion	Endoribonuclease	FIH1/HIF pathway	Suppress angiogenesis in NSCLC cell line and xenografts	[101]
miR-16/322/497/17	miRNA	VEGFR2	Inhibit tumor angiogenesis in a murine Lewis lung cancer model	[103]
miR-125b	miRNA	VE-cadherin	Inducing nonfunctional blood vessel formation to inhibit tumor growth	[104]
Multi-functional NPs	siRNA	VEGF	Inhibits tumor proliferation and angiogenesis in orthotopic NSCLC	[105]

This table summarizes the research on ncRNA-based anti-angiogenic therapeutics in lung cancer

Nucleic acid aptamers are small, single-stranded DNA or RNA molecules chemically synthesized for binding to a specific target. As a therapeutic tool, aptamers have several advantages, such as their small physical size and lack of immunogenicity, and are thus an invaluable targeted delivery carrier for siRNAs, miRNAs, and chemotherapeutic agents [95]. Lai et al. constructed two nucleolin aptamer-siRNA chimeras targeting snail family zinc finger 2 (SLUG) and neuropilin 1 (NRP1). Combined treatment with these two aptamer siRNAs significantly silenced SLUG and NRP1 expression in lung cancer cells, leading to specific inhibition of tumor invasion and angiogenesis [96]. Liao et al. constructed a bivalent cyclic RGD–siVEGFR2 conjugate delivery system that can silence VEGFR2 expression by targeting neovascularization in endothelial cells. Furthermore, biRGD–siVEGFR2 exhibited synergistic antitumor activity with apatinib in NSCLC, which may represent a new strategy for clinical NSCLC treatment [97]. Activation of CXCR4 and STAT3 results in the initiation of multiple signaling pathways, leading to metastasis and angiogenesis in lung cancer; thus, CXCR4 and STAT3 are potential targets for anti-angiogenic therapy [98]. Li et al. developed a fluorinated polymeric CXCR4 antagonist-stabilized perfluorocarbon (PFC) to deliver therapeutic siSTAT3. These nanoemulsions suppressed tumor proliferation and angiogenesis by simultaneously down-regulating CXCR4 and STAT3 in lung metastases [99].

Dicer, a type III cytoplasmic endoribonuclease, is required for the maturation of the vast majority of miRNAs [100]. Chen et al. generated miRNA-deficient tumors by knocking out Dicer1 only and found that the depletion of all miRNAs notably suppressed NSCLC angiogenesis. Mechanistic studies suggested that miRNAs promote tumor responses to hypoxia and angiogenesis by repressing FIH1, an inhibitor of HIF-1α [101]. Previous research has shown that malaria infection could suppress Lewis lung cancer cell proliferation by inducing innate and adaptive antitumor immune responses [102]. Yang et al. found that intratumoral injection of exosomes derived from the plasma of Plasmodium-infected mice significantly suppressed Lewis lung cancer growth. High levels of miR-16/322/497/17 were detected in exosomes isolated from the plasma of mice infected with Plasmodium, and up-regulated expression of these miRNAs in ECs corresponded with down-regulated expression of VEGFR2 [103]. This study helped to advance potential exosome-based anti-angiogenic tumor therapeutics.

Muramatsu et al. found that miR-125b inhibits EC tube formation by inhibiting the translation of VE-cadherin. Then, they used non-viral vectors composed of the cationic polymer polyethylenimine to package miR-125b and directly injected it into subcutaneous tumors. By targeting VE-cadherin, miR-125b induced the formation of non-functional blood vessels and inhibited tumor growth in vivo, which suggested a therapeutic potential for tumor angiogenesis [104]. Li et al. designed a therapeutic strategy against orthotopic NSCLC tumors that can intelligently co-deliver siVEGF and the chemotherapeutic etoposide (ETO) through multi-functional nanoparticles (NPs). Compared with monotherapy, combination therapy in orthotopic NSCLC causes more significant tumor growth and metastasis by simultaneously inhibiting tumor proliferation and angiogenesis [105]. Therefore, ncRNA-based anti-angiogenic therapy may be a promising strategy.

Circulating miRNAs may potentially act as biomarkers for predicting the response to anti-angiogenic therapy. M. Joerger’s study revealed that circulating miRNAs could predict the efficacy of angiogenic drugs combined with targeted therapies. Pretreatment circulating miRNA profiling indicated that the expression of 12 miRNAs was significantly associated with tumor shrinkage after bevacizumab/erlotinib treatment, with miR-665 being the strongest predictive marker. Moreover, miR-223 was related to time to progression (TTP) after bevacizumab/erlotinib treatment andafter second-line chemotherapy [106].

## Conclusion

Tumor metastasis and chemotherapeutic resistance lead to the poor prognosis of lung cancer. Current research suggests that ncRNAs are crucial players in tumorigenesis and tumor angiogenesis in lung cancer. Even though the function of ncRNAs has been determined in the tumor angiogenesis, less attention has been given to research on the clinical application of ncRNAs, suggesting that the requirement for ncRNA studies is unmet. Nucleic acid therapeutics targeting angiogenesis, such as modified siRNAs and miRNA mimics, are promising therapeutics for lung cancer. In conclusion, despite the emerging application of ncRNAs in tumor diagnosis and therapy and the challenges in this field, targeting ncRNAs is still an innovative and promising strategy to improve therapeutic outcomes for lung cancer patients.

## Data Availability

Not applicable.

## Abbreviations

NSCLC: Non-small cell lung cancer
VEGF: Vascular endothelial growth factor
VEGFR: Vascular endothelial growth factor receptor
ncRNAs: Non-coding RNAs
miRNAs: MicroRNAs
lncRNAs: Long non-coding RNAs
ECs: Endothelial cells
HIFs: Hypoxia-inducible factors
FDA: Food and Drug Administration
3' UTR: 3' Untranslated region
HUVECs: Human umbilical vein endothelial cells
LUAD: Lung adenocarcinoma
HGF: Hepatocyte growth factor
CP: Ceruloplasmin
PHD: Prolyl hydroxylase domain
MVs: Microvesicles
SCLC: Small cell lung cancer
CAFs: Cancer-associated fibroblasts
LUSC: Lung squamous cell carcinoma
CCAT1: Colon cancer-associated transcript 1
FABP5: Fatty acid binding protein 5
circRNA: Circular RNA
PTEN: Phosphatase and tensin homologue deleted on chromosome 10
HBE: Human bronchial epithelial
GAS5: Growth arrest-specific transcript 5
siRNAs: Small interfering RNAs
ASOs: Antisense oligonucleotides
SLUG: Snail family zinc finger 2
NRP1: Neuropilin 1
PFC: Perfluorocarbon
ETO: Etoposide
NPs: Nanoparticles
TTP: Time to progression
